# Clindamycin-resistant among *Staphylococcus aureus*: Investigation into phenotypic and genotypic profiles

**DOI:** 10.1371/journal.pone.0329467

**Published:** 2025-08-18

**Authors:** Mariyam Khursheed, Maryam Sulaiman, Abdulrahman M. Alhassan, Tameem H. Al Awad, Ahmed E. Ibrahim, Godwin Wilson, Hana A. Abdelrahaman, Nahla O. Eltai

**Affiliations:** 1 College of Health Sciences, Qatar University, Doha, Qatar; 2 Harvard American School, Doha, Qatar; 3 Glangwili Hospital, Wales, United Kingdom; 4 Hamad General Hospital, Doha, Qatar; 5 Biomedical Research Center, Qatar University, Doha, Qatar; Cairo University - Faculty of Pharmacy, EGYPT

## Abstract

**Background:**

Antimicrobial resistance (AMR), particularly methicillin-resistant *Staphylococcus aureus* (MRSA), is a pressing global health concern. These bacteria are increasingly becoming resistant to the most commonly available treatment options. As a choice, the macrolide lincosamide-streptogramin B (MLSB) is used, with clindamycin being the preferred drug. However, an alarming number of staphylococcal strains are developing resistance to MLSB. The resistance exhibits several phenotypes, including inducible MLSB (iMLSB), constitutive MLSB (cMLSB), and macrolide streptogramin B (MSB). One of the biggest challenges is the accurate detection of iMLSB in routine laboratory tests, as they appear erythromycin-resistant and clindamycin-sensitive unless the two antibiotics are placed adjacent to each other, which leads to clinical therapeutic failure.

**Method:**

To achieve this, double disc diffusion (D test) was used to test iMLSB phenotypically. In addition, the genetic determinants were identified through singleplex PCR using specific primers to detect *erm* (A, B, C) and *msr* genes associated with the different phenotypes of MLSB resistance.

**Result:**

Among 161 *S. aureus* isolates, 42 (26.1%) were erythromycin-resistant; 25 (15.5%) showed an iMLSB phenotype, and 16 (9.9%) displayed an MSB phenotype. One MRSA isolate expressing cMLSB phenotype. Genotypic analysis revealed a prevalence of *erm*C in 60% and *msr* in 40% of S. *aureus* isolates.

**Conclusion:**

The D-test is a reliable method for identifying inducible clindamycin resistance in clinical diagnostics, to support antibiotic use and treatment stewardship in Qatar.

## Introduction

Antimicrobial resistance (AMR) is a growing and pervasive threat to global public health, leading to severe illnesses, prolonged hospitalization, and highly increased healthcare expenses [1]. According to the Centers for Disease Control and Prevention (CDCP), approximately 23,000 deaths occur annually due to acquired diseases caused by AMR bacteria [2]. *Staphylococcus aureus* (*S. aureus)* is a major contributor to bacterial infections worldwide, ranging from superficial skin infections to life-threatening diseases [3,4]. It’s particularly concerning due to multidrug-resistant (MDR) characteristics, especially Methicillin-resistant *Staphylococcus aureus* (MRSA) which exhibit resistance to beta-lactam antibiotics and harbor the *mec*A or *mec*C gene [4,5]. In addition, it has been linked to a high mortality rate of 20,000 annual deaths recorded in the US [6]. In Qatar, a 2020 study found that 34% of infective endocarditis (IE) cases were caused by *Staphylococcus* [7].

However, another concern is resistance to macrolide-lincosamide-streptogramin B (MLSB), including erythromycin and clindamycin that belong to the macrolide and lincosamide classes, respectively [8–11]. The improper and excessive use of erythromycin has led to the development of several mechanisms responsible for several resistance phenotypes, including inducible MLSB (erythromycin resistant, clindamycin sensitive *in vitro*), constitutive MLSB (erythromycin resistant, clindamycin resistant), MSB phenotype (erythromycin resistant, clindamycin sensitive both *in vitro* and *in vivo*) [11,12]. iMLSB is particularly problematic because *S. aureus* appears sensitive to clindamycin in vitro, but can develop resistance during treatment with erythromycin, leading to clinical therapeutic failure, widespread resistance trend, and may impact the empirical treatment protocols that may involve alternative antibiotics [13,14]. The phenotype cMLSB is a mechanism where *S. aureus* is inherently resistant to clindamycin, regardless of environmental conditions or erythromycin presence [13]. These resistant phenotypes are driven by plasmid-borne *erm* genes (erythromycin ribosome methylase), which encode a methyltransferase that adds methyl groups to modify the ribosomal binding sites and prevent the antibiotics from effectively inhibiting the bacterial protein synthesis [11,15,16]. In the case of MSB phenotype, mediated by *msrA* genes that involve an efflux pump using an ATP-mediated protein transporter that expels erythromycin while maintaining sensitivity towards clindamycin [17–19]. Identifying these resistant phenotypes is crucial, especially in MRSA cases, as resistance mediated by the *erm* genes can be inducible and may not be detected by routine susceptibility tests unless specifically screened using the D-test. Failure to detect iMLSB may lead to treatment failure, emphasizing the importance of accurate detection and surveillance. According to communication with experts and professionals at HMC, clindamycin has been frequently reported to be effective in treating skin infections, especially in patients allergic to the β-lactams class of antibiotics.

Moreover, Qatar presents a unique epidemiological landscape due to its diverse, multicultural population, high influx of expatriates, and a healthcare system that interfaces with individuals from various geographical backgrounds. These factors contribute to the importation and potential dissemination of AMR determinants, including iMLSB. Despite the clinical importance, data on the prevalence and molecular characteristics of the iMLSB phenotype in the Gulf Cooperation Council GCC region remain scarce [20–23]. Our focus on a tertiary care hospital in Qatar (the main Health Care provider) allows us to generate foundational data that reflects both local resistance trends and the broader implications for the GCC Countries, where similar demographic and healthcare dynamics are present. Consequently, this study aims to assess the prevalence of the MLSB phenotype among clinical *S. aureus* isolates from patients at Hamad General Hospital (HGH), by implementing a routine D-test as a valuable diagnostic tool for detecting inducible clindamycin resistance. Therefore, it helps to improve antibiotic stewardship and treatment strategies in Qatar.

## Methodology

### Ethical clearance and analysis

Ethical approval was obtained from the Medical Research Centre at HGH, protocol no. MRC-01-20-1216 and the Institutional Biosafety Committee (IBC) at Qatar University approval number, QU-IBC-2023/022. The patient’s identifiers and demographic information were not collected. As such, each sample was given a serial number, and all patient identifiers were removed to maintain patient confidentiality. All the calculations were conducted using Microsoft Excel.

### Bacterial isolates

This study utilized a total of 161 *S. aureus* isolates, which were randomly collected from a range of clinical specimens without specification, obtained from patients admitted to Hamad General Hospital (HGH). The samples were processed and analyzed by the Microbiology Laboratory at the hospital as part of standard diagnostic protocols. No exclusion criteria were applied to the selection of isolates to ensure an unbiased representation of *S. aureus* strains circulating in the hospital setting. Samples were identified as *S. aureus* using MALDI-TOF (Bruker Daltonik GmbH, Bremen, Germany), according to the manufacturer’s protocol.

The study was designed as a cross-sectional investigation, with sample collection conducted retrospectively. The total of 161 isolates represents all available, non-duplicate *S. aureus* strains processed by the laboratory within the defined study period, ensuring both feasibility and statistical relevance while capturing a representative snapshot of the iMLSB phenotype. isolates were subsequently transported under refrigerated conditions (4°C) to the Biomedical Research Center (BRC) at Qatar University, where they were stored at −80°C until further analysis.

### Methicillin susceptibility determination

#### Disk diffusion test (Kirby-Bauer Test).

The obtained *S. aureus* isolates from the hospital were tested against cefoxitin using the disc diffusion method to differentiate MRSA isolates from MSSA. Briefly, a 0.5 McFarland suspension of an overnight culture of *S. aureus* isolates was prepared and swabbed on Muller-Hinton agar media (Himedia, Mumbai, India). Then, a single cefoxitin disc (30 μg) (Liofilchem®, Italy) was applied to the center of the plate and incubated at 37ºC for 18–24 hours. The zone of inhibition was measured and interpreted according to the CLSI guidelines 2020 [24]. Isolates with zones of inhibition (≤ 21 mm) were considered resistant (MRSA), and inhibition zones of (≥22 mm) were interpreted as susceptible (MSSA).

The quality control organisms used in this study included (MRSA) ATCC 25923 and MSSA S15 as positive controls and *Escherichia coli* (*E. coli*) ATCC 25922 as a negative control in all the experiments.

#### PCR amplification.

PCR amplification reactions were performed using the Hotstar Taq plus master mix kit (Qiagen, Germany) according to the manufacturer’s instructions and were used to identify and differentiate the MRSA and MSSA isolates. Two sets of primers were used: *mec*A forward: AAAATCGATGGTAAAGGTTGGC, *mec*A reverse: AGTTCTGGAGTACCGGATTTGC, and *spa* forward: TAAAGACGATCCTTCGGTGAGC, *spa* reverse: CAGCAGTAGTGCCGTTTGCTT, respectively. Biometra TAdvanced thermal cycler (Biometra, Göttingen, Germany) was used under the following conditions: initial denaturation at 95 °C for 10 min. Followed by a 40 PCR amplification cycle for the *mec*A gene and 35 for the *spa* gene, consisting of denaturation at 95 °C for 30 sec, annealing at 53 °C (*mec*A gene) and 50 °C (*spa* gene) for 30 sec, and extension at 72 °C for 1 min. The final extension was performed at 72 °C for 10 min [25]. Afterwards, 5 µl of PCR-amplified products were used in a 1.2% electrophoresis gel (Agarose- LE, Ambion®, USA) and visualized using the iBright CL1000 Imaging System (Thermo Fisher, USA). The amplification products of the mecA and *spa* genes were observed at 533 bp and 371 bp, respectively [25].

### Detection of MLSB resistance phenotypes

#### Erythromycin susceptibility test.

The disc diffusion test was conducted as previously described for cefoxitin, utilizing an erythromycin disc (15 μg) (Liofilchem, Italy). Isolates with a zone of inhibition ≥23 mm were classified as susceptible to erythromycin, while those with a zone diameter of ≤13 mm were classified as resistant according to CLSI 2020 [24]. Only erythromycin-resistant isolates were subsequently subjected to the D-test.

#### Double disc diffusion test (D test).

The double disc diffusion test (D test) was performed to identify iMLSB phenotype using erythromycin (15 μg) and clindamycin (2 μg) (Liofilchem, Italy) discs following CLSI recommendation [26].This time, erythromycin and clindamycin discs were placed approximately 15–20 mm apart on the inoculated plates and incubated at 37° C for 18–24 hrs. The resulting phenotypes of the D test were interpreted as follows:

- iMLSB phenotype: this phenotype shows resistance to erythromycin and sensitivity to clindamycin when tested separately. However, when these two antibiotics are placed adjacent to each other, a flattening D-shaped zone of inhibition around the clindamycin disc towards the erythromycin disc indicates the induced clindamycin resistance.
- cMLSB phenotype: exhibiting resistance to both erythromycin and clindamycin.
- M/MSB phenotype: demonstrating resistance to erythromycin but susceptibility to clindamycin, lacking the characteristic flattening towards the erythromycin disc.
- Sensitive (S) phenotype: displaying susceptibility to clindamycin and erythromycin [27].

### Detection of the genetic determinant of MLSB resistance

#### DNA extraction.

Genomic DNA was extracted from the erythromycin-resistant *S. aureus* isolates using the boiling method [28]. To accomplish the extraction, a few single colonies of the bacterial isolates were suspended in 500 µl of PBS and incubated in a thermo-shaker (Grant-bio, UK) at 100 ºC for 10 minutes. Then, the tubes containing the boilate were centrifuged for 5 minutes at 8000 rpm using a mini centrifuge (Thomas Scientific, Swedesboro), allowing the bacterial cell component to precipitate and the supernatant that contains the extracted DNA. The concentration and purity of the eluted DNA samples were assessed using a NanoDrop Lite Spectrophotometer (Thermo Fisher Scientific, USA). The results indicated adequate quality and yield suitable for PCR amplification. This method is widely recognized for its simplicity, cost-effectiveness, and rapid assessment of nucleic acid purity. DNA samples were subsequently stored at –20 °C until use in downstream molecular applications.

#### Conventional polymerase chain reaction (PCR).

The extracted DNA was used to prepare a conventional PCR mixture of each gene in a total reaction volume of 25 µl, containing 12 µl HotStar Taq Plus master mix (Qiagen, Germany), 1µl of forward and reverse primers of each *msr*, *erm*A*, erm*B*, and erm*C genes, Table 1 [29–31] 8 µl of nuclease-free water and 2 µl of Color Red reagent as loading dye. 5 µl of extracted genomic DNA was added separately for each tested sample. The reaction was amplified using a Biometra TAdvanced PCR machine (Analytik Jena, Germany) as in Table 1. After the amplification run, all PCR products were electrophoretically run on 1% agarose gel (AgaroseLE, Ambion®, USA) and visualized using the iBright™ CL1000 Imaging System (Thermo Fisher, US). The control used in this experiment was *Escherichia coli* ATCC 25922, as a negative control. *S. aureus* BAA-977, as a positive control for the *erm*A gene. *Enterococcus faecalis* ATCC 51299, as a positive control for *erm*B. Positive controls for detecting the *ermC* and *msr* genes were obtained from HMC as identified isolates.

**Table 1:** Primer sequences for *msr*, *erm*A, *erm*B, and *erm*C targeted genes and amplified product size.

Target gene	Primer sequence* (5′-3′)	PCR condition	PCR fragment size (bp)
*msr*	F: GGCACAATAA GAGTGTTTAA AGG	95ºC for 10 minutes 35 cycles of (94°C for 30 seconds, 50ºC for 30 seconds, 72ºC for 30 seconds) 72ºC for 1 minute	190 [29]
	R: AAGTTATATC ATGAATAGAT TGTCCTGTT		
*erm*A	F: GTTCAAGAAC AATCAATACA GAG	95ºC for 10 minutes 30 cycles of (94° C for 30 seconds, 50ºC for 30 seconds, 72ºC for 30 seconds) 72ºC for 5 minutes	421 [29]
	R: GGATCAGGAA AAGGACATTT TAC		
*erm*B	F:CCGTTTACGAAATTGGAACAGGTAAAGGGC	95ºC for 10 minutes 30 cycles of (94° C for 30 seconds, 55ºC for 30 seconds, 72ºC for 30 seconds) 72ºC for 5 minutes	142 [30]
	R: GAATCGAGACTTGAGTGTGC		
*erm*C	F: ATCTTTGAAATCGGCTCAGG	95ºC for 10 minutes 30 cycles of (94° C for 30 seconds, 50ºC for 30 seconds, 72ºC for 30 seconds) 72ºC for 5 minutes	295 [31]
	R: CAAACCCGTATTCCACGATT		

*All lyophilized primer sequences were purchased from IDT.

## Results

### MRSA and MSSA isolates identification

Overall, n = 116 (72%) of the 161 obtained isolates were resistant to cefoxitin and recorded as MRSA, while n = 45 (27.9%) were sensitive to cefoxitin and verified as MSSA.

### Detection of MLSB resistance phenotypes

#### Erythromycin susceptibility test.

Of 161 *S. aureus* isolates, 42 (26%) were reported as erythromycin-resistant, while 119 (73.9%) were sensitive. Among the 42 isolates, 31 (73.8%) were MRSA, and 11 (26%) were MSSA.. The percentage of erythromycin-resistant among MSSA was 11/45 (24.4%) and among MRSA was 31/116 (26.7%).

#### Double disc diffusion test, D test.

**Fig 1:**
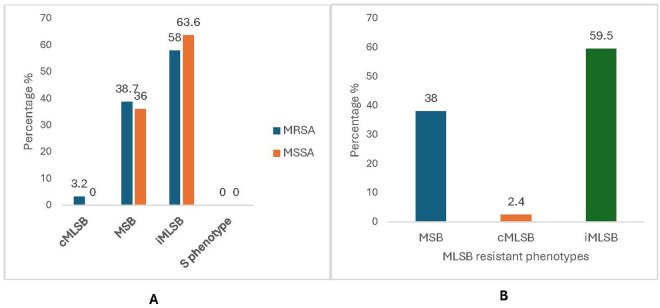
Phenotypic prevalence of MLSB phenotypes A) Percentage of *S. aureus* isolates that express MLSB-resistant phenotypes. B) MLSB-resistant phenotypes in percentages among MRSA and MSSA isolates. cMLSB = constitutive Macrolide Lincosamide Streptogramin B. iMLSB = inducible Macrolide Lincosamide Streptogramin B. MSB = Macrolide Lincosamide Streptogramin B.

**Fig 2:**
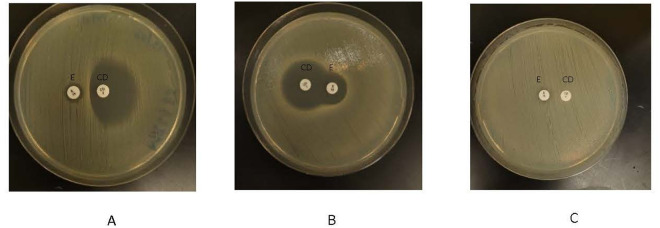
Phenotypic profile of macrolide-lincosamide-streptogramin B using double disc diffusion test. (A) D test positive, inducible clindamycin resistance (iMLSB phenotype). (B) D test negative, Macrolide Stretogramin B resistance (MSB phenotype). (C) D test negative, Constitutive clindamycin resistance (cMLSB phenotype). E = Erythromycin, CD = Clindamycin.

Of 42 erythromycin-resistant isolates, 25 (59.5%) were D test positives, while the rest were D test negative Fig 1 (A). Among MRSA isolates, 1 (3.2%) expressed cMLSB phenotype, 12 (38.7%) expressed MSB phenotype, and 18(58%) expressed iMLSB phenotype, with no detection of S phenotype. While for MSSA isolates, 4(36%) showed the MSB phenotype, and 7(63.6%) expressed the iMLSB phenotype, with no report of cMLSB or S phenotype, Figs 1(B) and 2.

### Genotypic detection of *msr* and *erm*A, *erm*B, and *erm*C genes

#### Conventional polymerase chain reaction (PCR).

**Fig 3:**
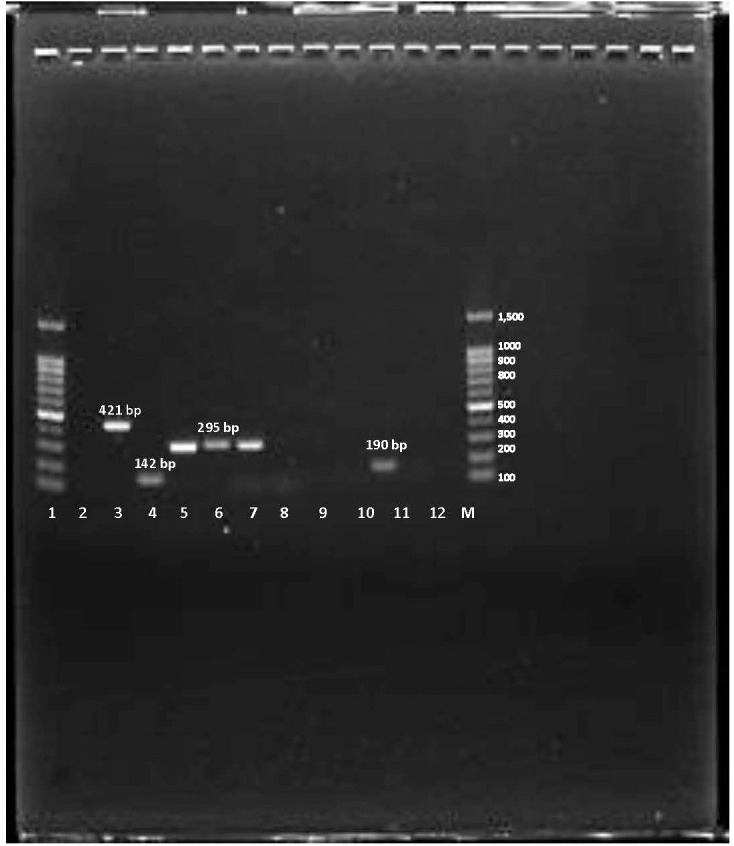
Representative graph for *msr*, *erm*A, *erm*B, and *erm*C encoding for MLSB. Lane (1), *E. coli* ATCC 25922 as negative control. Lane (2) *S. aureus* BAA-977 as positive control for *erm*A. Lane (3) *E. faecalis* ATCC 51299 as a positive control for the *erm*B gene. Lane (4), positive control for *erm*C. Lane (5-6) positive samples for *erm*C. Lane (7-9) negative samples. Lane (10) positive control for the *msr* gene. Lane (11-12) negative samples. Negative samples for the *msr* gene. M, molecular size DNA ladder (100 bp).

Among the 42 isolates, the most commonly detected gene was *ermC,* found in 25 isolates (60%), followed by *msr* in 17 isolates (40%). Neither *erm*A nor *erm*B genes were detected in Fig. 3. Of the MRSA isolates, 18(58%) harbored the *ermC gene*, while 13 carried the *msr* gene. For MSSA strains, 7 isolates harbored the *ermC* gene and 4 carried the *msr* gene, as in Table 2.

**Table 2:** Genotypic results of *msr, erm*A*, erm*B*,* and *erm*C using conventional PCR among MRSA and MSSA isolates.

Gene name	MRSA^e^	MSSA^f^	Total
n = 31	n = 11	n = 42
number, (%)	number, (%)	number, (%)
***msr*** ^**a**^	13, (42%)	4, (36%)	17, (40%)
***erm*A** ^**b**^	0	0	0
***erm*B** ^**c**^	0	0	0
***erm*C** ^**d**^	18, (58%)	7, (64%)	25, (60%)

a Methionine sulphoxide reductase.

b Erythromycin resistance methylase A.

c Erythromycin resistance methylase B.

d Erythromycin resistance methylase C.

e Methicillin Resistant *Staphylococcus aureus.*

f MSSA = Methicillin Sensitive *Staphylococcus aureus*.

## Discussion

The rising prevalence of MDR and the emergence of MRSA strains, for which there are limited treatment options, have posed significant challenges in managing staphylococcal infections [32]. As a result, there has been an increased use of MLSB antibiotics to treat these infections. In particular, clindamycin has the advantages of low-cost, minimal side effects, and good tissue penetration [33,34]. Additionally, it features an anaerobic cover, antitoxin effect, and immunomodulator. Our study found that a higher proportion of MRSA isolates (26.7%) exhibited resistance to erythromycin than MSSA isolates (24.4%), consistent with results reported from Spain, where the erythromycin resistance was three times more prevalent in MRSA isolates compared to MSSA isolates [35]. Similarly, a study conducted in South India reported that a higher proportion of MRSA isolates exhibited erythromycin resistance than MSSA isolates [36]. This indicates a higher consumption and use of the erythromycin antibiotic worldwide [37]. MRSA is known to exhibit increased resistance to more antibiotic agents, such as linezolid, vancomycin, teicoplanin, and daptomycin, compared to the MSSA strain [38]. Upon performing the D-test, we observed that the iMLSB-resistant phenotype was more ubiquitous in MRSA isolates compared to MSSA isolates, in line with findings from a study conducted in India [39]. To the best of our knowledge, up until 2024, there was no published data on inducible clindamycin resistance among *S. aureus* isolates in Qatar. However, a recent study by our group reported that 23% of community-associated MRSA strains exhibited the iMLSB phenotype [40]. However, only a few regional studies displayed a higher iMLSB in MSSA than in MRSA, which highlights the need for more region-specific studies to accurately assess the prevalence of iMLSB resistance among MSSA [20–23]. Still, in Bangalore, a study reported a higher prevalence of iMLSB among MSSA-isolated strains from clinical samples, although the sample size was small [41]. Interestingly, a recent study in Iran concluded a 96% of the MRSA isolates from cockroaches displayed the iMLSB phenotype, which plays a significant role in the dissemination of resistance factors as mechanical vectors [42]. The variable resistance findings can be attributed to the geographical locations of the patient population included. It is also worth noting that iMLSB was the predominant phenotype in our study, followed by MSB and then cMLSB. A study conducted in Serbia reported similar phenotypic patterns, with iMLSB being the most frequent phenotype and cMLSB being the least frequently occurring phenotype [43]. This likely enables the bacteria to avoid the fitness cost associated with constitutive resistance while surviving erythromycin exposure. Additionally, factors like clinical treatment regimens may contribute to the greater prevalence of iMLSB in *S. aureus* populations. In contrast, the phenotypic distributions in a study by Zachariah and his colleagues differed considerably from our findings, with the MSB phenotype being more prevalent than iMLSB [44]. A similar phenotypic pattern was also observed in studies conducted in Nigeria, Uruguay, and Egypt [45–48]. The variations in these outcomes may be attributed to varying macrolide and lincosamide prescription rates and consumption across different geographical regions. Also, many other elements, such as the difference in the inclusivity of inpatients and outpatients, hospital and community-acquired infection, and the disease prevalence among different age groups (children or adults), could contribute to both similarities and variations observed [47].

The genotypic analysis in this study helped pinpoint two essential genes responsible for resistance mechanisms leading to distinct phenotypes, namely the *erm* and *msr* genes. Our study findings demonstrate a clear correlation between the phenotypic detection of MLSB phenotypes and the genotypic findings, so a higher prevalence of iMLSB is associated with a high prevalence of *erm*C gene expression. Similarly, the MSB phenotype is predominantly linked to the *ms*r gene, which is reported to be the second most prevalent gene found. Among the different *erm* genes, *erm*C was the only one detected in MRSA and MSSA isolates. This study’s exclusive presence of *ermC* may indicate a more specific and focused mechanism for inducible clindamycin resistance. The high prevalence of the *ermC* gene in our *S. aureus* isolates can be attributed to the geographical distribution influenced by the local pattern of antibiotic usage. Our isolates were randomly collected from clinical specimens at a tertiary care hospital, which may represent a specific patient population with prior antibiotic exposure, particularly macrolides and lincosamides. Such exposure may exert selective pressure favoring *ermC*-harbouring strains. Moreover, the *erm*C gene is commonly located on a plasmid, which facilitates the horizontal transfer of the gene among *S. aureus* in the population and contributes to its predominance [49]. Further investigation is warranted to explore this aspect in greater depth, as this is considered preliminary data on this area. Additionally, PCR results indicate the presence of the *msr* gene among the MRSA and MSSA isolates by expressing the MSB phenotype (i.e., the gene encoding antibiotic efflux pumps). Similar to our findings, other studies have also observed this [50], most notably the one conducted in Iran, in which a majority of the isolates harbored the *erm*C gene [29,51]. On the contrary, certain studies observed a more significant proportion of *erm*A genes among MRSA isolates [48]. Also, a survey at Mansoura University Children’s Hospital in Egypt revealed a more substantial proportion of the *erm*A gene [52]. Another study from Iran found a variable prevalence of all MLSB-related genes A, B, and C isolated from the nasal carriage of healthcare workers [53]. The discrepancies and inconsistencies in genotype prevalence across various countries can be attributed to several factors, such as antibiotic usage and policies in each country, genetic factors, diagnostic practices, and public health initiatives [54,55]. In numerous studies, the presence of the *msr* gene is consistently significantly lower than the *erm* genes, underscoring the geographical dependency of genotype prevalence [56]. Therefore, monitoring the incidence of iMLSB is crucial for guiding stewardship programs and treating staphylococcal infections. This can be effectively achieved through routine antimicrobial susceptibility testing (AST) in laboratories, mainly using the D-test, to assess the appropriate use of clindamycin and erythromycin.

## Conclusion

The D-test is a crucial and reliable tool for detecting inducible clindamycin resistance in *S. aureus.* Its use in routine antimicrobial susceptibility testing allows for precisely identifying the iMLSB phenotype, which is decisive for guiding appropriate antibiotic medication. Distinguishing inducible resistance assists clinicians in avoiding treatment failures, particularly in regions with high iMLSB, and supports the judicious use of clindamycin and erythromycin. As part of effective antibiotic stewardship, the D-test is vital in optimizing treatment outcomes and combating the mounting challenge of antimicrobial resistance.
